# Hering’s Analytical Therapeutics*American Homœopathic Publishing Company. 2d ed.

**Published:** 1882-06

**Authors:** P. P. Wells


### HERING'S ANALYTICAL THERAPEUTICS: MIND AND DISPOSITION.*

Charles Lamb began an essay by saying, "of books that are *not* books, the first I shall mention is Josephus' History of the Jews." We reverse this and say, of books which *are* books we would first mention that the title of which is at the head of this review.

In these days, when the utterances of the kingly maker of proverbs is being so fearfully verified: "That of making many books there is no end," it is a rare experience when we can take in our

*American Homœopathic Publishing Company. 2d ed.

hand a new one, made with a worthy and valuable purpose, in which this purpose is fully attained.

The motives impelling bookmakers are various; some more and some less worthy. Some apparently write for reputation; that the world may know they are in it. Some for money; that they may be the better able to live in it. Some—and they are comparatively few—write to give utterance and form to knowledge they possess, that the world may be made the better and happier by its diffusion. These write because they cannot help it. Our late loved and honored colleague, the author of the *Analytical Therapeutics*, was of this small number. He wrote because he was overflowing with the gathered fruits of a long life of incessant labor, and he could not choose but give it to the world that they might also know. In the volume before us he has given this in a fullness and strength altogether admirable. The author had given the best part of his life to the study and enlargement of our materia medica. While he possessed of other knowledge more than most men, of materia medica he was pre-eminently *the* master. Of this, scientifically and practically he was full. Full even to overflowing—the outcome of which, in part, was the volume before us.

The objective of the author in his preparation of this volume is found in this sentence, from his introduction: "If the arduous labor bestowed for years upon this work should render efficient aid to all who are earnestly trying to heal the sick; if it should enable them to select, in most cases intrusted to their care, the proper drug, the author will be richly repaid for all his exertions." To help others in difficult and responsible duties, this was the objective of the author. And who, of all who ever knew him while living, will not say how just like him this object is: always helping others, and apparently most happy when helping most.

Let it be remembered always that this objective was *a help* to the labors of others, and never and in no sense was it intended to be *a substitute* for such labor. To make the work of finding the specific easier, not in any way is it a pretence of superseding the necessity of that work. To lighten the burdens, not to dispense with them. To enable the practitioner to find his specific for himself, not to find it for him.

The importance of "the symptoms of the mind" as indicators of the specific curative, in *all* cases of sickness was clearly understood by our author, and their authority recognized by him in his long and busy practical life. He was at the same time fully aware of the very general neglect of these in the general practice of the many in our school, who, imperceptibly to themselves, fall into habits of routine in daily duties, having found the difficulty of seeking curatives through the guidance of *all* the symptoms, too great for their patience or strength. It was in part the objective of our author to strengthen these feeble knees, or what would be an equivalent of this, to lighten the burdens of such as far as these were found in the needful search for the simillimum of this most important class of symptoms, this he has attempted by bringing into a beautiful order of arrangement, these symptoms as they have been developed in provings and by clinical experience in connection with disturbances in the functions of the organs of the body, simultaneously with those of the mind; so presenting in the clearest manner and light the pathogenetic relations of mental and bodily symptoms, and through this relationship to point more clearly the way of search for the curative and to enable the prescriber the more certainly to recognize this when found. The motive of the author will be appreciated by many who, without this help, have struggled with the task this was intended to lighten. It is enough to characterize the work after stating its objective, to name its author—HERING. Hering the teacher, the observer, the philosopher, the laborious, careful and truthful recorder of facts; the never-tiring worker, the model physician, the true and noble-hearted man.

It is not too much to say of him that for this work he possessed qualifications belonging to no other man. The breadth and vigor of grasp of his mental powers, of his unequaled knowledge of materia medica, the gathered fruit of his life-work, his wonderful powers of ready analysis and comparison, of his habits of incessant toil, his thorough conviction of the truth of the homœopathic law and of the immense interest humanity has in its truth, and that this interest hangs wholly on a thorough and truthful understanding of the materia medica, and his clear perception of the difficulties in the way of him who will strive for this. These and the many more qualities of heart and mind, and the varied knowledge which made up the working man whom we knew, fitted him more than any other man was ever fitted, to give to the world this most valuable and important volume. When we say of this, that in its execution the work is worthy of its author we speak but the truth. When this is said no higher praise can be given it. We congratulate the practitioners of our school that they so readily appreciated the value of this work, as to render so soon the publication of a second edition a necessity. P. P. W.

